# ECM1 modified HF-MSCs targeting HSC attenuate liver cirrhosis by inhibiting the TGF-β/Smad signaling pathway

**DOI:** 10.1038/s41420-022-00846-4

**Published:** 2022-02-08

**Authors:** Qi Liu, Chengqian Lv, Qianqian Huang, Lei Zhao, Xiaoli Sun, Dandan Ning, Jingyang Liu, Yanan Jiang, Shizhu Jin

**Affiliations:** 1 Department of Gastroenterology and Hepatology, The Second Affiliated Hospital of Harbin Medical University, Harbin, Heilongjiang Province 150086 P. R. China; 2 Department of Pharmacology (State-Province Key Laboratories of Biomedicine-Pharmaceutics of China, Key Laboratory of Cardiovascular Research, Ministry of Education), College of Pharmacy of Harbin Medical University, Harbin, Heilongjiang Province 150081 P. R. China; 3 Translational Medicine Research and Cooperation Center of Northern China, Heilongjiang Academy of Medical Sciences, Harbin, Heilongjiang Province 150086 P. R. China

**Keywords:** Stem cells, Gastrointestinal diseases

## Abstract

Hair follicle-derived mesenchymal stem cells (HF-MSCs) show considerable therapeutic potential for liver cirrhosis (LC). To improve the effectiveness of naïve HF-MSC treatments on LC, we used bioinformatic tools to identify an exogenous gene targeting HSCs among the differentially expressed genes (DEGs) in LC to modify HF-MSCs. Extracellular matrix protein 1 (ECM1) was identified as a DEG that was significantly downregulated in the cirrhotic liver. Then, ECM1-overexpressing HF-MSCs (ECM1-HF-MSCs) were transplanted into mice with LC to explore the effectiveness and correlated mechanism of gene-overexpressing HF-MSCs on LC. The results showed that ECM1-HF-MSCs significantly improved liver function and liver pathological injury in LC after cell therapy relative to the other treatment groups. Moreover, we found that ECM1-HF-MSCs homed to the injured liver and expressed the hepatocyte-specific surface markers ALB, CK18, and AFP. In addition, hepatic stellate cell (HSC) activation was significantly inhibited in the cell treatment groups in vivo and in vitro, especially in the ECM1-HF-MSC group. Additionally, TGF-β/Smad signal inhibition was the most significant in the ECM1-HF-MSC group in vivo and in vitro. The findings indicate that the genetic modification of HF-MSCs with bioinformatic tools may provide a broad perspective for precision treatment of LC.

## Introduction

Liver cirrhosis (LC) is induced by a variety of causes, including infectious factors, chemical injury, and metabolic and genetic factors. There is no effective way to cure LC except for orthotopic liver transplantation (OLT). However, most patients with cirrhosis do not have the opportunity to receive OLT due to a lack of financial resources, immune rejection, or shortage of donor livers, among other reasons [1–4]. Considering that the excessive deposition of extracellular matrix (ECM) caused by hepatic stellate cell (HSC) activation is a critical aspect in the course of LC development [5–8], more effective strategies targeting HSCs to treat LC are urgently needed.

The emerging application of mesenchymal stem cells (MSCs) provides a promising method for treating end-stage hepatic disease. Hair follicle mesenchymal stem cells (HF-MSCs) are derived from the bulge of hair follicles [9] and present broad application prospects considering their characteristics of rich source materials, easy access, low immunogenicity, and almost no limitation according to age [10]. In addition, the extraction of HF-MSCs is not as traumatic as bone marrow-derived mesenchymal stem cells (BM-MSCs), and more importantly, HF-MSCs show greater proliferation than BM-MSCs [11]. HF-MSCs have been proven to have the ability to differentiate into tissue-specific cells such as fat, bone, cartilage, smooth muscle cells, neurogliocytes, melanocytes, and hepatocytes [12–15], which indicates that HF-MSCs may have extensive prospects for liver disease treatment. Despite the above advantages, the role of naïve MSCs in LC treatment remains to be improved [16, 17], and the overexpression of genes in MSCs has proven to be a more effective way to treat liver fibrosis/cirrhosis [18–22].

High-throughput sequencing and gene chips provide the ability to elucidate the changes in genetic information during the development of liver diseases. Here, we used bioinformatic tools to identify an HSC-related target gene among the differentially expressed genes (DEGs) in LC.

Extracellular matrix protein 1 (ECM1) was identified as a DEG downregulated in LC for the modification of HF-MSCs. ECM1 is a secreted glycoprotein and is involved in embryonic chondrogenesis, skin differentiation, angiogenesis, and cell proliferation [23–27]. ECM1 is mainly secreted by hepatocytes and contributes to the maintenance of hepatic homeostasis. The amount of ECM1 produced by hepatocytes is reduced when liver fibrosis occurs, while exogenous ECM1 supplementation can reverse hepatofibrosis by blocking HSC activation [28]. Therefore, increasing the level of ECM1 continuously and steadily in the damaged liver may become a new strategy to reverse LC.

In our study, we applied ECM1-overexpressing HF-MSCs in LC to evaluate the effectiveness and mechanism of gene-transfected HF-MSCs, which may provide a relevant theoretical basis for the application of ECM1-HF-MSCs in LC treatment.

## Materials and methods

### Identification of DEGs from microarray data

**Fig. 1:**
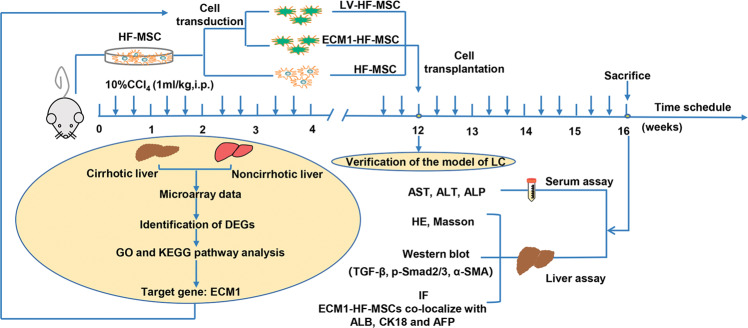
Overview of the experimental design. The DEGs related to LC were analyzed using bioinformatic tools. ECM1 was selected from the DEGs and was then used to modify HF-MSCs. The cells were injected into mice with CCl_4_-induced LC through the tail vein. Four weeks after cell transplantation, serum and liver tissue were collected for HE and Masson staining and Western blotting and immunofluorescence detection.

The whole experimental design is shown in Fig. 1. The gene expression profile GSE103580 dataset was downloaded from the GEO database (http://www.ncbi.nlm.nih.gov/geo). The GSE103580 dataset contains 86 samples, including 67 cirrhotic samples and 19 noncirrhotic samples. The online analysis software GEO2R (http://www.ncbi.nlm.nih.gov/geo/geo2r) was used to analyze the DEGs between the cirrhotic group and the noncirrhotic group, according to a screening threshold of *P* < 0.05 and a fold change ≥1.5. Volcano plots and heat maps generated by the R language packages ggpubr and pheatmap were used to display the screened DEGs.

### Gene ontology (GO) and kyoto encyclopedia of genes and genomes (KEGG) pathway enrichment analysis of DEGs

To analyze the function of DEGs in LC, GO analysis and KEGG pathway enrichment analysis of DEGs were performed using the online Database for Annotation, Visualization, and Integrated Discovery (DAVID, version 6.8; david.ncifcrf.gov) tool, and the enrichment difference was regarded as significant when *p* < 0.05. The analysis results are shown in bubble charts using the R language package ggplot.

### Protein–protein interaction network of DEGs encoding proteins

To visualize the proteins encoded by DEGs, the STRING database (http://string-db.org; version 10.0) was used to construct a PPI network with a confidence of 0.40. Then, the filtered PPI results were imported into Cytoscape 3.7.2 software to construct a visual molecular interaction network.

### Experimental animals

C57BL/6J (specific pathogen-free) male mice were used in this experiment. Seven- to nine-day-old mice were chosen to extract primary HF-MSCs, and six- to seven-week-old mice weighing 18–22 g were selected for the subsequent experiments. The mice were all from the scientific research center of the Second Affiliated Hospital of Harbin Medical University. The mice could obtain feed without intervention at the scientific research center. All animal experimental protocols were conducted in accordance with the principles of medical ethics and approved by the Ethics Committee of the Second Affiliated Hospital of Harbin Medical University (NO. SYDW2019-240).

Our pre-experimental results show that the difference was statistically significant when the minimum sample size was 6. Mice that died during the experiment were excluded from the experiment, and the surviving mice were used in subsequent experiments. No blinding was done due to the obvious grouping basis by experimenter.

### Isolation and culture of primary HF-MSCs

Healthy mice were anesthetized, and the skin around their beard was disinfected with medical alcohol. Then, the skin around the beard was resected and minced into soybean-sized pieces. Type I collagenase (0.1%, Sigma–Aldrich, St Louis, MO, USA) was used to hydrolyze the collagen components of connective tissue at 4 °C. After 13 h, the digestion reaction was stopped by the addition of fetal bovine serum (FBS, ScienCell, USA), and the collagenase attached to the tissues was removed with PBS. The hair follicles were peeled off with microtweezers under a binocular microscope within one hour. Then, one hair follicle was inoculated in a cell culture plate containing DMEM/F12 (Gibco, Gaithersburg, MD, USA), 15% FBS (ScienCell), and 1% penicillin-streptomycin (Gibco). Cells were passaged when they reached 70–80% confluence approximately 12–14 days later. Second- or third-generation HF-MSCs with a good growth status were used for subsequent experiments. All operations were performed in a sterile environment.

### Identification of HF-MSCs

Third-generation HF-MSCs were used for phenotyping analysis. The cells (1 × 10^6^ cells per specimen) were incubated with FITC-, PE-, PerCP-, and APC-labeled monoclonal antibodies against CD29, CD90, CD43, and CD31 for half an hour at 4 °C without light. The antibodies were then washed off with PBS. The phenotypes were analyzed by a FACSCanto II flow cytometer (BD Biosciences, USA).

The multi-differentiation potential of the HF-MSCs was determined by assessing their ability to differentiate into adipocytes and osteocytes. Alizarin red (Sigma–Aldrich) staining was used to detect mineralized nodules in osteogenic differentiated cells, and the lipid droplets in the differentiated cells were detected by Oil red O (Sigma-Aldrich) staining after adipogenic induction according to the instructions.

### Lentivirus transfection of HF-MSCs

Lentiviral vectors (LVs) were used to carry the target gene ECM1 to infect HF-MSCs to achieve lasting expression of ECM1. LVs (GOSL0219611) were purchased from Shanghai Gene Chemical Company, including one blank LV encoding only green fluorescent protein (GFP) (GFP-LV) and one ECM1-overexpressing LV encoding both GFP and the ECM1 protein (ECM1-LV). The blank LV was used for the preliminary experiment to achieve the optimal multiplicity of infection (MOI). Third-generation HF-MSCs were inoculated into 96-well plates for 24 h until the cells filled 30% of each well. The HF-MSCs were then infected with GFP-LV (LV-HF-MSCs) at different MOIs (20, 40, 60, 80, and 100). After 48 h, the fluorescence intensity and transfection efficiency were detected to obtain the optimal MOI. Then, the HF-MSCs were transfected with ECM1-LV at the optimal MOI for 72 h for subsequent experiments.

### Animal model of LC and experimental design

To induce the mouse LC model, 10% carbon tetrachloride (CCl_4_, diluted in oil) was injected into the abdominal cavity every Monday and Thursday, 1 ml/kg each time. After 12 weeks, the LC model was verified according to liver biochemical indices and liver pathology. Then, the model mice were divided into 4 groups at random (based random order generator), and administered different treatments via the tail vein: (1) the HF-MSC group was injected with 1 × 10^6^ HF-MSCs (*n* = 6); (2) the LV-HF-MSC group was injected with 1 × 10^6^ LV-HF-MSCs (*n* = 6); (3) the ECM1-HF-MSC group was injected with 1 × 10^6^ ECM1-HF-MSCs (*n* = 6); and (4) the model group was injected with an equivalent amount of saline (*n* = 6). Healthy mice (*n* = 6) were intraperitoneally injected with the same volume of saline. Four weeks after cell therapy, the liver tissue and serum of the sacrificed mice were collected for the subsequent analysis. Immunofluorescence was used to estimate the homing and differentiation of the transplanted cells in host liver tissues. Serum analysis was used to evaluate the recovery of liver function. Histological and molecular biological analyses were used to evaluate pathological recovery and the changes in pathway-related proteins.

### Activation of hepatic stellate cells and transwell assays

The murine HSC cell line JS1 was purchased from Otwo Biotech (ShenZhen, China) and stimulated with TGF-β1 [29] (Cat 100-21, 5 ng/ml, Peprotech, USA) for 48 h. Transwell chambers with an aperture of 0.4 µm (Corning, USA) placed on a 6‐well plate were used to coculture HF-MSCs with activated JS1 cells. The cell concentration of the two kinds of cells was 2.5 × 10^5^ cells/ml after resuspension. Next, 1.5 ml of an HF-MSC suspension was added to the upper chamber, and 2.6 ml of a JS1 cell suspension was added to the lower chamber. After coculture with HF-MSCs for 48 h, JS1 cells were collected for protein analysis.

### Western blot analysis

Proteins that had been extracted from cells and liver tissue on ice with lysis solution were centrifuged. The supernatant was collected for concentration measurements and boiled for preservation or for subsequent experiments. The same amount of protein from each group was added to an SDS-PAGE gel and subjected to electrophoresis. The protein in the SDS-PAGE gel was transferred to a polyvinylidene fluoride (PVDF) membrane (Millipore, USA). Then, the membrane was blocked with 5% skimmed milk for 1.5 h at room temperature and incubated with primary antibodies (anti-ECM1 antibody, 11521-1-AP, 1:1000, Proteintech; anti-TGF-β1, #3711, 1:1500, Cell Signaling Technology; anti-α-SMA, sc-53142, 1:2000, Santa Cruz Biotechnology; anti-p-Smad2/3, AF3367, 1:1000, Affinity; β-actin, ab8226, 1:1000, Abcam) at 4 °C overnight. Thereafter, the membrane was further incubated with the corresponding secondary antibody (ab205718, 1:10000, Abcam) at 37 °C for 50 min. Enhanced chemiluminescence (ECL) solution was used to visualize protein bands, and the relative protein level was calculated based on the housekeeping gene content with ImageJ.

### Immunofluorescence staining

OCT-embedded liver sections held in an ultralow-temperature refrigerator were cut to a thickness of 6 µm each and were left at room temperature for half an hour, rinsed with TBST to remove OTC on the surface and then fixed with cold acetone (−20 °C). An appropriate amount of goat blocking serum was used to block the liver sections for an hour. Primary antibodies (anti-CK18, ab181597, 1:200, Abcam; anti-ALB, ab207327, 1:500, Abcam; anti-AFP, ab213328, 1:200, Abcam) were used to bind specific proteins on the sections at 4 °C overnight. The liver slices were then subsequently incubated with the secondary antibody (SA00013-4, 1:500, Proteintech) for an hour at 37 °C. The nuclei were stained with DAPI for 4 min. After adding antifade mounting medium (Beyotime, China), the staining of the liver slices was observed under a fluorescence microscope (Olympus, Japan).

### Pathological analysis

The tissue sections were successively transferred to different concentrations of xylene and ethanol for dewaxing and dyed in hematoxylin. Then the sections were rinsed with flowing water until they turned blue and dyed in eosin solution for 2 min. Thereafter, the stained sections were dehydrated with ethanol and treated with xylene. The transparent sections were dripped with neutral gum and sealed with a cover glass.

The paraffin sections were dewaxed and then successively washed with tap water and distilled water. The sections were subsequently stained with Weigert’s iron hematoxylin staining solution for 5 min. After differentiation with an acidic ethanol solution, Masson blue solution was used to restore the blue coloration. Then the sections were stained with Lichun red magenta staining solution for 5 min, and the slices were sealed with neutral gum. Images of the slices were obtained with a BX51 microscope (Olympus, Japan).

### Analysis of liver biochemical indices in serum

Blood samples of all groups were taken from the left ventricle for serological analysis when the mice were sacrificed. The obtained blood was placed in an EP tube and centrifuged. The supernatant was stored in an ultralow temperature freezer or used for subsequent experiments directly. The levels of alanine aminotransferase (ALT) (Cusabio, China), aspartate aminotransferase (AST) (Abcam), and alkaline phosphatase (ALP) (Cloud-Clone Corp, Wuhan, China) were measured according to the instructions to evaluate the liver function of each group.

### Statistical analyses

At least three independent replicates were performed for each experiment. All the data are presented as the means ± SD, and charts were generated using GraphPad Prism 8.0 (GraphPad Prism Software, San Diego, CA, USA). Data from more than two groups were analyzed using one-way analysis of variance (ANOVA), followed by Tukey’s test, and two-tailed Student’s *t* test was used for comparisons between two groups. *F* test was used to determine whether two population variances are equal. Non-parametric test was used for data that does not satisfy the normal distribution. *P* < 0.05 was considered statistically significant.

## Results

### DEGs identification

**Fig. 2:**
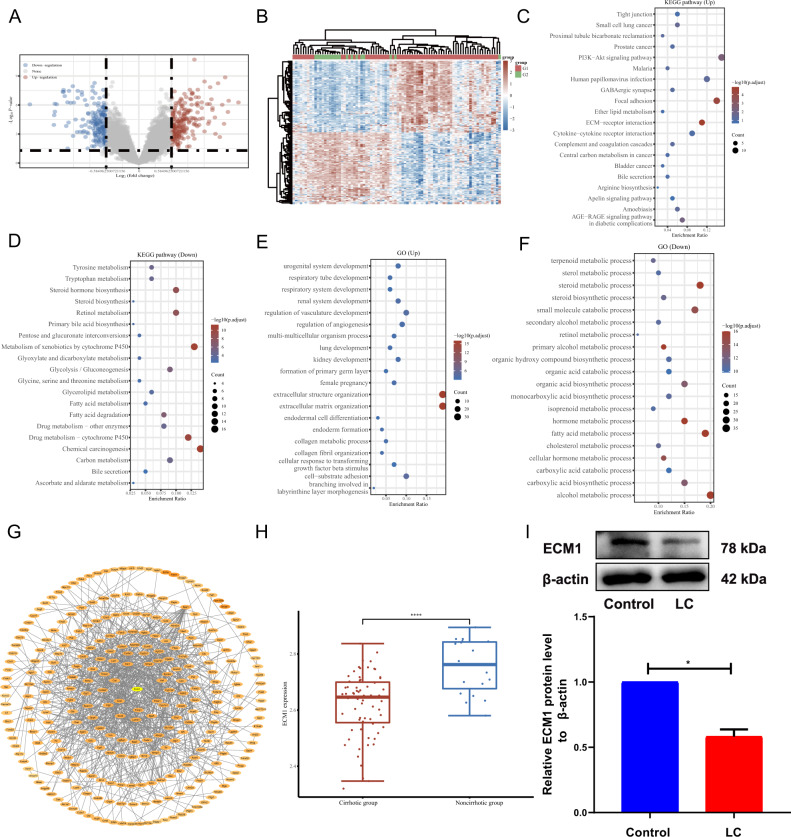
DEG analysis and identification of ECM1 as the target gene. **A** Volcano plot of DEGs in GSE103580. **B** Heat map of DEGs in GSE103580. G1 represents the cirrhotic group; G2 represents the noncirrhotic group. Red indicates upregulation; green represents downregulation. KEGG pathway analysis of upregulated DEGs (**C**) and downregulated DEGs (**D**). GO enrichment of upregulated DEGs (**E**) and downregulated DEGs (**F**). **G** Protein–protein interaction networks among the DEGs. **H** Expression of ECM1 in DEGs. **I** Western blotting analysis and semiquantitative analysis of ECM1 expression in the control group and the model group. Data are shown as the means ± SDs (*p < 0.05, **p < 0.01, ***p < 0.001).

After normalizing the profile GSE103580 dataset, a total of 377 DEGs were obtained, 178 (47.21%) of which were downregulated in LC, while 199 (52.79%) were upregulated with a cutoff criterion of *P* < 0.05 and a fold change ≥1.5. Volcano plots and heat maps were used to present the screened DEGs (Fig. 2A, B).

### Identification of ECM1 as a target gene for HF-MSC transfection

GO and KEGG analyses were used to enrich the biological processes and functions of DEGs. The DEGs were primarily enriched in pathways such as ECM-receptor interaction, focal adhesion, and chemical carcinogenesis (Fig. 2C, D). From what GO analysis represented (Fig. 2E, F), we observed that the DEGs were mainly related to the upregulation of extracellular structure organization and ECM organization. Considering that ECM plays a crucial role in LC, we took this as the starting point to identify foreign genes and aim to reduce ECM deposition.

Next, for the visualization of all DEG-encoded proteins, the online tools STRING and Cytoscape software were used to perform PPI network analysis of the DEGs. A total of 280 nodes and 847 edges in the aggregate were obtained in Cytoscape. In this network, we found the ECM1 gene, which is abundantly expressed in the ECM, was one of the DEGs downregulated in LC (Fig. 2G, H). Afterwards, we verified that ECM1 expression was decreased in LC mice relative to the healthy mice in vivo (Fig. 2I). Study also have shown that ECM1 can inhibit HSC activation [28]. Based on the above evidence, we choose ECM1 as the target gene for transfecting HF-MSCs.

### Identification of HF-MSCs and target gene expression in ECM1-HF-MSCs

**Fig. 3:**
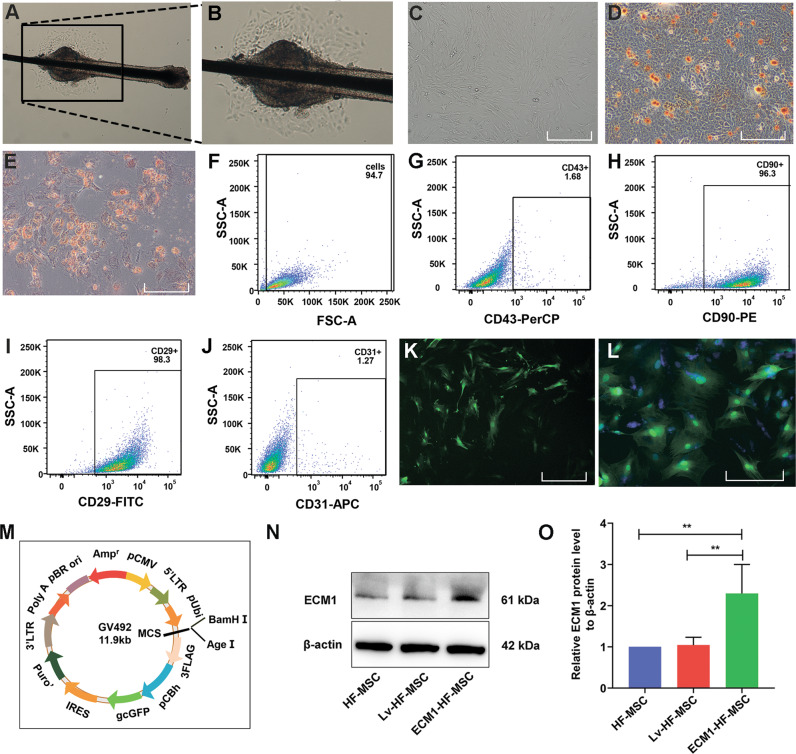
Identification and transfection of HF-MSCs. Primary (**A**, **B**) and P3 (**C**) HF-MSCs. **D**, **E** Adipogenic and osteogenic differentiation of HF-MSCs. **F**–**J** Identification of specific antigenic markers in HF-MSCs by flow cytometry. **K** The transfected HF-MSCs show green fluorescence under a 4X microscope. **L** HF-MSCs expressing GFP were costained for nuclear detection and observed under a 10X microscope. **M** The constructed lentiviral vector containing ECM1 and GFP. **N**, **O** Immunoblotting analysis and semiquantitative analysis of ECM1 in the HF-MSC group, the LV-HF-MSC group, and the ECM1-HF-MSC group. Data are shown as the means ± SDs (*p < 0.05, **p < 0.01, ***p < 0.001). Scale bar = 50 μm.

Primary HF-MSCs migrated across the culture plate and aggregated around the hair follicle bulge on the fourth day (Fig. 3A, B). As the cells proliferated, the third-generation HF-MSCs fused into a monolayer and grew in a spindle shape (Fig. 3C). Oil red O staining and alizarin red staining confirmed that the HF-MSCs had the potential to differentiate into adipocytes and osteocytes (Fig. 3D, E). FACS analysis showed that the cell surface CD90 and CD29 (antigen phenotype of MSCs) expression was highest, while <2% of HF-MSCs expressed CD43 (hematopoietic stem cell marker) and CD31 (endothelial cell surface marker) (Fig. 3F–J). The above results indicated that most of the cells that we extracted were HF-MSCs, with high purity. The preliminary results showed that the transfection efficiency of HF-MSCs was highest when the MOI was 40 (Fig. 3K). HF-MSCs that were GFP labeled and DAPI stained after infection at an optimal MOI of 40 are shown in Fig. 3L. A schematic diagram of the constructed lentiviral vector containing GFP, the target gene ECM1, and the puromycin resistance gene is shown in Fig. 3M. ECM1 was shown to be overexpressed in ECM1-HF-MSCs relative to HF-MSCs and LV-HF-MSCs in qualitative and semiquantitative analyses (Fig. 3N, O).

### Verification of the LC model in mice

**Fig. 4:**
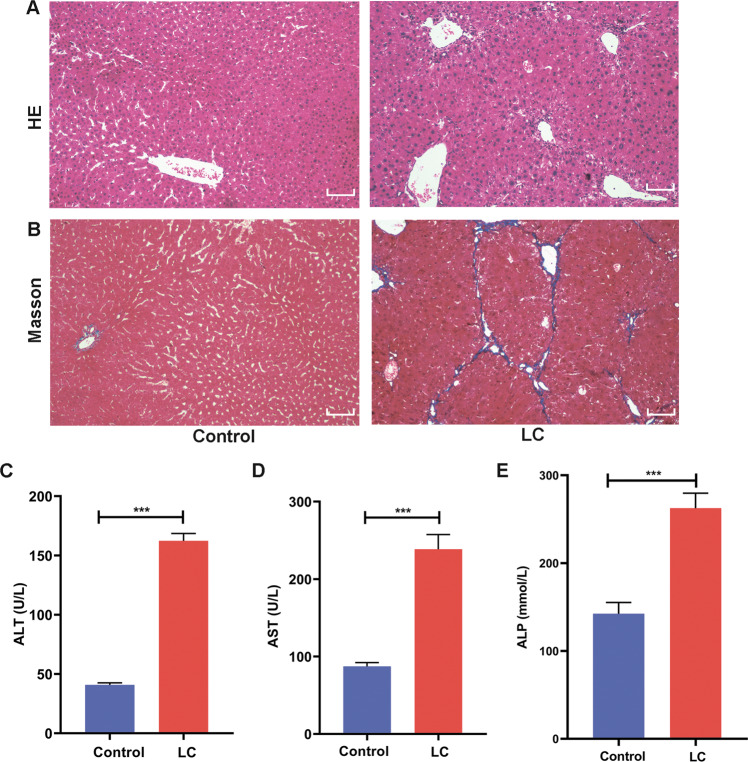
Verification of the LC model. **A**, **B** HE staining and Masson staining in the control group and the LC group. **C**, **D**, **E** Comparison of the liver serological indices of ALT, AST, and ALP in the control group and the LC group. Scale bar = 50 μm.

In the 12th week after the injection of CCl_4_, liver pathology and liver biochemical indices were used to evaluate the model establishment. The staining of the pathological section shows that there was no obvious abnormality detected in the heathy mice, while in the LC group, there was a degeneration and necrosis of hepatocytes, with a large area exhibiting pseudolobule structure. Additionally, large amounts of fibrous tissues proliferated and were connected with each other, and these tissues separated and wrapped the liver tissue to form pseudolobules (Fig. 4A, B). In addition, after induction by CCl_4_, AST, ALT and ALP levels were several times higher than those in healthy mice (Fig. 4C–E). These results indicated that a 12-week modeling cycle can lead to LC in mice.

### ECM1-HF-MSCs inhibit the activation of HSCs by blocking the TGF-β/Smad pathway in vitro

**Fig. 5:**
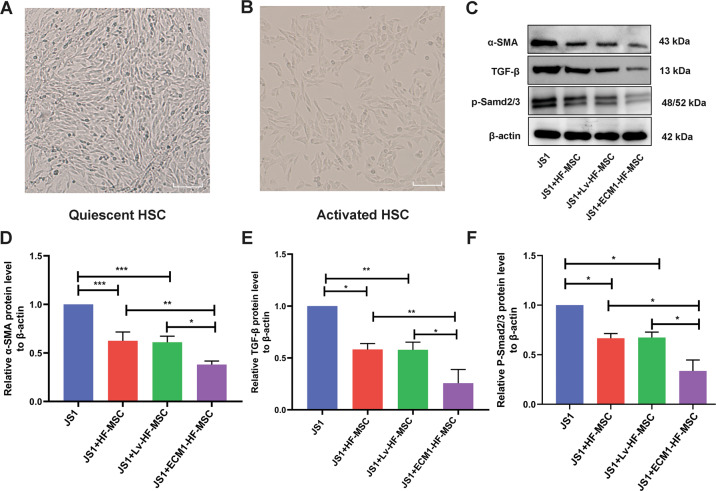
ECM1-HF-MSCs inhibit the TGF-β/Smad signaling pathway and activation of HSCs in vitro. **A** The quiescent murine hepatic stellate cell line JS1. **B** Activated JS1 cells. Scale bar  = 50  μm. **C** Western blotting analysis of a-SMA, TGF-β, and p-Smad2/3 in all groups. **D**–**F** Semiquantitative analysis of the pathway proteins and a-SMA. Data are shown as the means ± SDs (*p  <  0.05, **p  <  0.01, ***p  < 0.001).

The unactivated JS1 cells were polygonal under an inverted phase-contrast microscope (Fig. 5A). After 48 h of TGF-β1 stimulation [29], the activated JS1 cells showed a long fusiform shape (Fig. 5B) and were used for subsequent experiments. To detect the activation of JS1 cells and changes in pathway proteins, JS1 cells were collected after coculture with HF-MSCs, LV-HF-MSCs, and ECM1-HF-MSCs for 48 h. The a-SMA expression was suppressed in cell therapy groups in contrast to the JS1 group, while the expression in the ECM1-HF-MSC group was the lowest. Significantly decreased TGF-β1 and p-Smad2/3 levels were observed in the treatment groups, and the expression level was the lowest in the ECM1-HF-MSC group (Fig. 5C–G). These results indicated that ECM1-HF-MSCs have a greater ability than naïve HF-MSCs to inhibit the activation of JS1 cells through the TGF-β/Smad pathway.

### Grafted HF-MSCs exist in the liver and express hepatocyte-specific surface markers

**Fig. 6:**
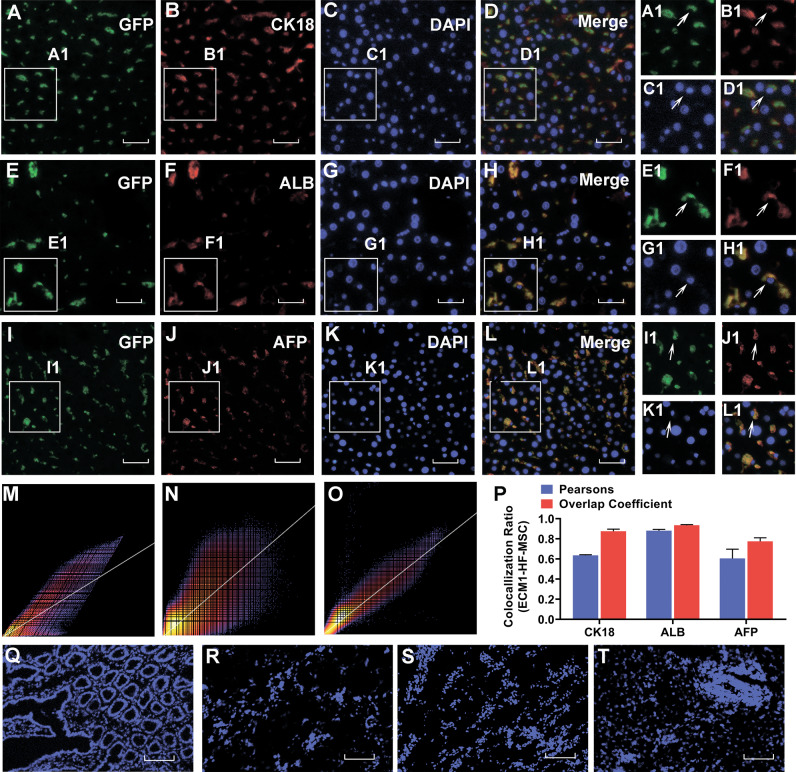
ECM1-overexpressing HF-MSCs home to the injured liver site and differentiate into hepatocyte-like cells. Colocalization of GFP-expressing ECM1-HF-MSCs and the hepatic-specific surface markers CK18 **A**–**D**, ALB **E**–**H**, and AFP **I**–**L. A1**–**L1** Magnification of regions of (**A**–**L**). **M**–**O** Scatter plots of GFP-expressing ECM1-HF-MSCs and CK18, ALB, and AFP levels. **P** Pearson’s correlation and the overlap coefficient of liver sections costained for CK18, ALB, and AFP in the ECM1-HF-MSC group. **Q**–**T** GFP-labeled ECM1-HF-MSCs were rarely observed in the intestine, kidney, lung, or spleen. Scale bar (**A**–**L**), 50 μm; Scale bar (**Q**–**T**), 200 μm.

In the 4th week after ECM1-HF-MSC transplantation, we detected the distribution of GFP-stained HF-MSCs in the liver, intestine, kidney, lung and spleen by immunofluorescence. As shown in Fig. 6A, E, I, HF-MSCs were detected in the injured liver but were barely observable in the intestine, kidney, lung, and spleen (Fig. 6Q–T). In addition, we found that the transplanted GFP-labeled ECM1-HF-MSCs were costained with CK18, ALB, and AFP, which are hepatocyte-specific surface markers (Fig. 6B, F, J). It indicated that ECM1-HF-MSCs differentiated into hepatocyte-like cells (HLCs) (Fig. 6D, H, L). Pearson’s correlation, overlap coefficient and scatter plot, which reflect the degree of the colocalization of GFP and hepatocyte-specific surface markers, are shown in Fig. 6M–P.

### Transplantation of ECM1-HF-MSCs show a better effect on promoting injured liver repair and improving liver function

**Fig. 7:**
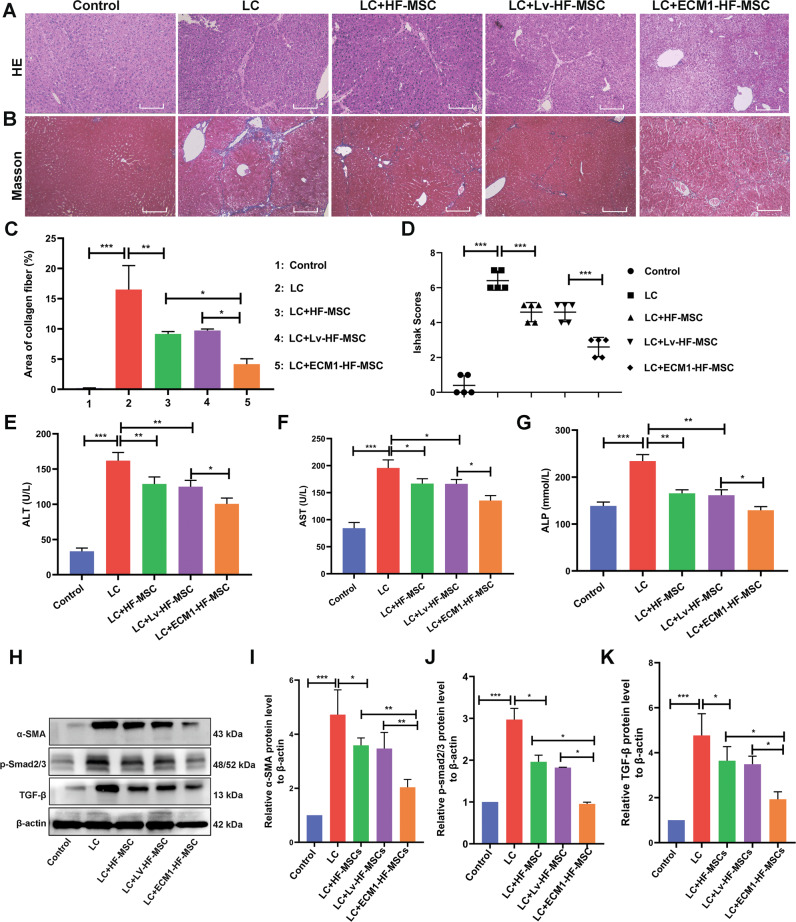
ECM1-HF-MSCs show a better effect in LC treatment through inhibiting the activation of HSCs and the TGF-β/Smad signaling pathway in vivo. **A**, **B** HE and Masson staining in each group. Scale bar = 100  μm. **C** Area of collagen fibers in each group. **D** Ishak scores show the degree of LC in all groups. **E**–**G** Changes in the serological indices of ALT, AST and ALP. **H**–**K** Western blotting and semiquantitative analysis of a-SMA, p-Smad2/3, and TGF-β in all groups. Data are shown as the means ± SDs (*p  <  0.05, **p <  0.01, ***p  <  0.001).

HE and Masson staining were used to evaluate liver pathological changes after cell treatments. It showed that the lobules and portal areas were normal in the control group, while large areas of proliferative fibrous tissue divided and wrapped the hepatocyte regeneration nodules into pseudolobules of different sizes in the model group. However, the typical pathological features of cirrhosis were significantly improved after cell therapy, of which the most significant improvement was achieved by the administration of ECM1-HF-MSCs (Fig. 7A, B). In addition, the area of collagen fibers was reduced by cell treatment compared with that in the LC mice, while that in the ECM1-HF-MSC group was smallest (Fig. 7C). The Ishak score was used to detect damage to liver tissue in each group. The model group showed the most serious damage, and the ECM1-HF-MSC group showed the most obvious remission (Fig. 7D).

A serological assay was used to detect liver function in each group. The high levels of AST, ALT and ALP in the LC mice were reversed by cell treatment and showed different downward trends. It is worth mentioning that the serological indices in the ECM1-HF-MSC group tended to be normal (Fig. 7E–G). The above results showed that ECM1-HF-MSCs can improve CCl_4_-induced LC more effectively than other treatments in terms of both pathology and liver function.

### ECM1-transfected HF-MSCs inhibit the activation of HSCs via the TGF-β/Smad pathway in vivo

We detected HSC activation at the protein level in vivo. The α-SMA level was remarkably inhibited by cell treatment in comparison with the model group, and the inhibition was most significant in the ECM1-HF-MSC group (Fig. 7H, I).

The remarkable tendency of high TGF-β and p-Smad2/3 expression observed in the LC model was reversed by cell transplantation treatment. The TGF-β and p-smad2/3 levels in liver tissue among the treatment groups showed marked differences, and the protein levels in the ECM1-HF MSC group presented a more downward trend than other treatment groups (Fig. 7H, J, K). This finding indicated that the overexpression of ECM1 in HF-MSCs resulted in greater inhibition of HSC activation via the TGF-β/Smad pathway than naïve HF-MSCs.

## Discussion

In this study, we used bioinformatic tools to screen the HSC-related target gene ECM1 from the DEGs identified in LC and then transfected it into HF-MSCs. We found that ECM1-transfected HF-MSCs represented a more effective treatment for LC in terms of restoring pathology and liver function than naïve HF-MSCs, which may be mediated by multiple mechanisms.

The LC model was established with CCl_4_, and its characteristics were very similar to the pathological characteristics of human drug-induced LC [30]. The method of CCl_4_ application in our research was intraperitoneal injection, which results in a high modeling rate and survival rate, with good reproducibility [31]. Twelve weeks after the intraperitoneal injection of CCl_4_, the mouse liver pathological and serological analysis showed that typical pseudolobules formed, and the serum indices were significantly increased, which indicated that we successfully established the LC model in mice.

Cirrhosis often leads to death due to various complications, and an effective treatment is currently lacking. MSC transplantation has been shown to improve liver fibrosis, even in the advanced stages of LC [5, 32]. In addition, MSCs are a good vector that can be easily transfected with retroviruses and lentiviruses, with transfection rates of 50–80% [33], and these retroviruses and lentiviruses are commonly used for the overexpression of foreign transgenes [34]. We transfected ECM1 into HF-MSCs with lentivirus to overexpress ECM1. After cell transplantation, we found that liver function was improved and that the content of collagen fibers was decreased by cell therapy, especially by ECM1-HF-MSC treatment.

Multiple mechanisms may be involved in the therapeutic effect of ECM1-HF-MSCs on LC. This effect requires a sufficient number of MSCs to home to the injured tissue and exert anti-inflammatory or differentiation effects to repair injured tissues [35]. SDF-1 is a widely studied chemokine involved in homing. When tissue damage occurs, SDF-1 expression in damaged cells increases, and MSCs are then recruited via the chemoattraction imposed by an SDF-1 concentration gradient and are retained at the injured site, which exhibits a high SDF concentration [36]. It shows that BM-MSCs can home to damaged tissues and survive in these tissues for up to 13 months [37, 38]. Similarly, we found that GFP-labeled ECM1-HF-MSCs existed in the damaged liver tissue 4 weeks after cell transplantation. However, we only detected the homing of ECM1-HF-MSCs in the 4th week after cell transplantation. The homing efficiency of ECM1-HF-MSCs at different times can be further investigated in subsequent studies, and more effective methods for enhancing the chemotaxis of MSCs to the damaged liver can be explored.

Previous studies suggest that MSCs can differentiate into HLCs regardless of their origin [39–41], which can restore hepatocyte vitality when liver injury occurs. In our research, ECM1-HF-MSCs labeled with GFP in liver tissue expressed the hepatocyte-specific markers CK18, ALB and AFP, which indicated that ECM1-transfected HF-MSCs show a tendency to differentiate into HLCs. It requires strict conditions and the participation of a variety of cytokines for MSCs to different into hepatocytes. In addition, the Wnt pathway and epigenetic modifications also contributed to this process [42, 43]. However, the therapeutic effect of MSCs on LC is affected by the differentiation efficiency of MSCs [4]. More strategies to improve the differentiation efficiency of HF-MSCs need to be studied.

Next, we studied the effect of ECM1-HF-MSCs on the fate of HSCs. In the case of liver injury, HSCs with a dormant phenotype become myofibroblasts, which are the main source of ECM components in pathological fibrous tissue [44]. Yu F et al. [45] proved that MSCs inhibit HSC activation after coculture with MSCs in vitro. Here, we also found that HF-MSCs could reverse the activation of HSCs, while ECM1-HF-MSCs exerted a stronger inhibitory effect on HSCs. The effect of MSCs on HSCs could be realized by direct contact between the two kinds of cells or by transmitting signals to HSCs through cytokines secreted by MSCs to change the fate of HSCs. On the other hand, MSCs can exert indirect effects on HSCs by acting on immune cells, the immune response was reduced due to the immunosuppressive properties of MSCs, thereby reducing the pro-fibrotic stimulation of immune cells to HSCs [46]. It is widely accepted that the MSCs anti-fibrotic effect is mainly due to the paracrine factors [47, 48], the specific mechanism of HF-MSCs on HSCs deserves further exploration.

**Fig. 8:**
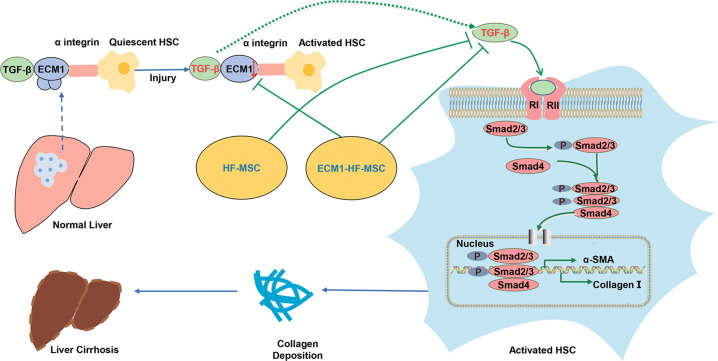
Overview diagram describing the mechanism of the inhibitory effect of HF-MSCs on HSCs. In a healthy liver, ECM1 interacts with αV integrin to maintain the latent state of TGF-β. After liver injury, ECM1 levels are decreased, leading to the activation of TGF-β by α_V_ integrin, which in turn activates HSCs through the TGF-β/Smad pathway. ECM1-HF-MSCs can inhibit the activation of TGF-β through exogenous supplementation with ECM1, which in turn affects the activation of HSCs, thereby alleviating LC.

Liver fibrosis and cirrhosis develop under the action of common signaling pathways, although the etiology of liver disease may be different. TGF-β1/Smad pathway activation is essential in liver fibrosis [49, 50]. Research proved that MSCs can alleviate LC by regulating the TGF-β1/Smad pathway [51], which is in line with our results. TGF-β exists as a latent complex in the liver, and αv integrin plays a role in interacting with latent TGF-β to convert it into activated TGF-β, leading to the development of LC [52, 53]. As shown in Fig. 8, ECM1 interacts with α_v_ integrin, which disturbs TGF-β activation to maintain liver homeostasis [54]. When ECM1 is reduced due to liver injury, TGF-β is activated, and the activation of HSCs is initiated. The trend is reversed when exogenous ECM1 is administered [28]. In our study, ECM1-HF-MSCs continuously supplied exogenous ECM1, thereby stably inhibiting the activation of TGF-β and HSCs. This explains why TGF-β1 and p-Smad2/3 levels in the ECM1-HF-MSC group were the lowest among all cell treatment groups.

In summary, this study showed that ECM1 modified HF-MSCs have the potential to migrate to the injured liver and differentiate in to HLCs. ECM1-HF-MSCs showed a more significant therapeutic effect on LC than naïve HF-MSCs, which may be mediated most likely by the inhibition of HSC pathological activation via the TGF-β/Smad pathway. Our research may provide evidence for the combination of bioinformatic technology and genetic engineering to achieve precise treatment of LC.

## Data Availability

The data that support the findings of this study are available on request from the corresponding author.
